# Image calibration in choroidal vascularity index measurement

**DOI:** 10.1186/s40942-023-00503-7

**Published:** 2023-11-10

**Authors:** Mehrdad Motamed Shariati, Nasser Shoeibi

**Affiliations:** Eye research center, Mashhad University of Medical Sciences, Mashhad, Iran

**Keywords:** Image processing, ImageJ software, Image calibration

## Abstract

Choroid is a tissue with a very high blood flow which is a metabolic supporter of the retina. Recently, the study of choroidal blood flow in ocular and systemic disorders is a hot topic in scientific research. With the advent of enhanced depth imaging OCT (EDI-OCT), it is possible to measure the entire choroidal thickness. The choroidal vascularity index (CVI) is a relatively new index in studying choroidal hemodynamics. However, the CVI measurement needs image processing. Image calibration is a necessary step before any image processing with software such as ImageJ.

We are writing to commend the authors on their valuable contribution to the field of retinal research through their article titled “Choroidal structure investigated by choroidal vascularity index in patients with inherited retinal diseases,“ which was published in the International Journal of Retina and Vitreous on 28 March 2023 [1]. The study addresses an essential aspect of ocular health, and the authors’ use of ImageJ software and the binarization method for choroidal vasculature analysis is commendable. However, we wish to bring to your attention a potential measurement error in the study that could impact the accuracy of the reported results.

The investigation into choroidal vascularity index in patients with inherited retinal diseases is an intriguing and novel area of research. The findings presented by Bayat et al. provide valuable insights into choroidal structure and its potential associations with inherited retinal diseases. The study’s clinical relevance cannot be overstated, as such insights could have implications for developing targeted therapeutic interventions to improve patient outcomes.

The authors’ choice to employ ImageJ software and the binarization method for choroidal vasculature analysis reflects a robust methodological approach [2, 3]. By utilizing well-established tools, they have ensured the credibility and reliability of their findings, which enhances the significance of the study within the scientific community.

However, upon examining Fig. 1 of the original article, we noticed a potential error in measuring the total choroidal area (TCA) and luminal area (LA) of the choroid. The authors measured the TCA within an area of 3000 microns in length and as wide as the thickness of the choroid, defining it as the region of interest (ROI). Our concern arises from the calibration of the image, specifically regarding the scale representation in the OCT image.

In Fig. 1A of the original article, it is evident that the scale of the OCT image is different in the vertical and horizontal dimensions (Fig. 1). Calibration of the image is crucial for determining the pixel-to-length ratio and setting the scale correctly [4]. It appears that the authors used the horizontal guide-line to set the scale, which may have resulted in the overestimation of the choroidal thickness and subsequently affected the measurements of TCA and choroidal LA.

**Fig. 1:**
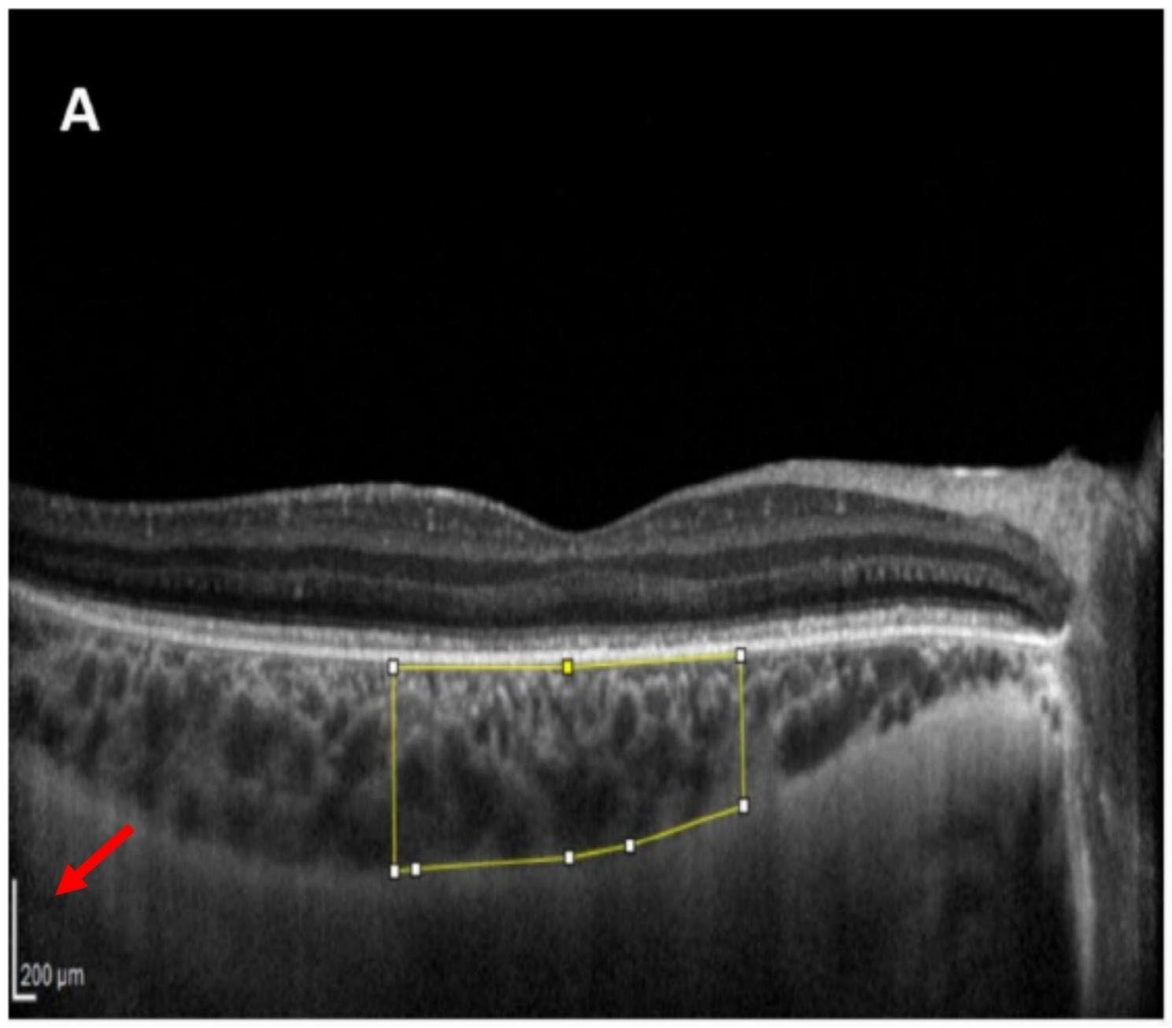
This the Fig. 1A of the original article. As shown with the red arrow, the scale of the represented OCT image is different vertically and horizontally

An example of the potential impact of this measurement error can be seen in the reported mean TCA of 2.49 mm² for healthy participants in Table 2 of the original article. Considering the defined region of interest with a lateral length of 3000 microns and usual choroidal thickness of 300 microns in the subfoveal region as the thickest choroidal region, this value appears anomalously high and prompts the consideration of potential calibration discrepancies.

## Calibrating the primary EDI-OCT image

**Fig. 2:**
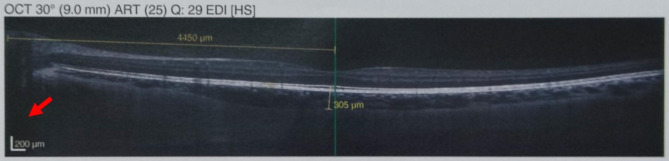
The red arrow shows that the scale is equal horizontally and vertically

The objective of image calibration before using the ImageJ software is to make the horizontal and vertical length scales equal. As shown in Fig. 2, to achieve this goal, the operator must set the length scale to 1:1 after image acquisition. After this step, the image will be ready for analysis with the ImageJ software.

While acknowledging the valuable work of Bayat et al., it is essential to note that measurement errors, particularly those related to calibration and scale determination, can inadvertently occur in scientific research. Similar studies analyzing images or quantitative data may also face challenges in ensuring precise measurements, underscoring the significance of addressing this issue.

## Data Availability

Not applicable.

## Abbreviations

CVI: Choroidal Vascularity index
LA: Luminal area
TCA: Total Choroidal Area
